# Bovine Gamma Delta T Cells Contribute to Exacerbated IL-17 Production in Response to Co-Infection with Bovine RSV and *Mannheimia haemolytica*

**DOI:** 10.1371/journal.pone.0151083

**Published:** 2016-03-04

**Authors:** Jodi L. McGill, Rachel A. Rusk, Mariana Guerra-Maupome, Robert E. Briggs, Randy E. Sacco

**Affiliations:** 1 Department of Diagnostic Medicine/Pathobiology, College of Veterinary Medicine, Kansas State University, Manhattan, Kansas, United States of America; 2 Pathobiology Graduate Program, Department of Diagnostic Medicine/Pathobiology, College of Veterinary Medicine, Kansas State University, Manhattan, Kansas, United States of America; 3 Ruminant Diseases and Immunology Research Unit, National Animal Disease Center, Agricultural Research Service, Ames, Iowa, United States of America; University of Iowa, UNITED STATES

## Abstract

Human respiratory syncytial virus (HRSV) is a leading cause of severe lower respiratory tract infection in children under five years of age. IL-17 and Th17 responses are increased in children infected with HRSV and have been implicated in both protective and pathogenic roles during infection. Bovine RSV (BRSV) is genetically closely related to HRSV and is a leading cause of severe respiratory infections in young cattle. While BRSV infection in the calf parallels many aspects of human infection with HRSV, IL-17 and Th17 responses have not been studied in the bovine. Here we demonstrate that calves infected with BRSV express significant levels of IL-17, IL-21 and IL-22; and both CD4 T cells and γδ T cells contribute to this response. In addition to causing significant morbidity from uncomplicated infections, BRSV infection also contributes to the development of bovine respiratory disease complex (BRDC), a leading cause of morbidity in both beef and dairy cattle. BRDC is caused by a primary viral infection, followed by secondary bacterial pneumonia by pathogens such as *Mannheimia haemolytica*. Here, we demonstrate that *in vivo* infection with *M*. *haemolytica* results in increased expression of IL-17, IL-21 and IL-22. We have also developed an *in vitro* model of BRDC and show that co-infection of PBMC with BRSV followed by *M*. *haemolytica* leads to significantly exacerbated IL-17 production, which is primarily mediated by IL-17-producing γδ T cells. Together, our results demonstrate that calves, like humans, mount a robust IL-17 response during RSV infection; and suggest a previously unrecognized role for IL-17 and γδ T cells in the pathogenesis of BRDC.

## Introduction

Human respiratory syncytial virus (HRSV) is a leading cause of acute lower respiratory tract disease in human infants worldwide. It is estimated that over 33 million children under the age of 5 years old contract the disease, resulting in over 3 million hospitalizations and nearly 200,000 deaths [1]. Currently, no licensed vaccine is available for HRSV.

Bovine respiratory syncytial virus (BRSV) is genetically and antigenically closely related to HRSV. BRSV infection is among the leading causes of lower respiratory tract infections in young calves [2–4]. BRSV infection in the calf parallels many features of HRSV infection in humans, including age-dependent susceptibility, microscopic lesions and innate and adaptive immune responses [3, 5, 6]. BRSV infection in neonatal calves represents a fully permissive, natural host-pathogen interaction that closely mimics HRSV infection in human infants. Thus, BRSV infection is increasingly recognized as an excellent model for studying disease pathogenesis and immunity to HRSV infection in children, as well as for pre-clinical testing of novel vaccines and therapeutics [3, 5, 6].

In addition to causing morbidity in calves due to uncomplicated infections, BRSV is an important contributing factor in the development of bovine respiratory disease complex (BRDC), a multifactorial disease condition affecting cattle in all stages and categories of production [2–4, 7]. The etiology of BRDC is not well understood but usually affects animals that are stressed due to weaning, shipping or comingling, and is a result of co-infections by multiple viral and bacterial agents, including BRSV, *Mannheimia haemolytica*, *Pasteurella multocida* and *Histophilus somni* [8–13]. BRDC is the leading cause of morbidity and mortality among feedlot cattle and is the most common cause of weaned dairy heifer mortality [3, 4]. Economic costs to the cattle industry due to BRDC have been estimated as high as $3 billion annually due to death losses, reduced performance and costs of vaccinations and treatment modalities [14]. Features of BRDC include significant pro-inflammatory cytokine production and bronchopneumonia including severe neutrophilia in the lungs. The mechanisms of immune suppression and disease pathogenesis contributing to the development of BRDC remain unclear.

Hallmarks of severe RSV infection in infants and BRSV infection in calves include rapid neutrophil infiltration, excessive mucus production and a delayed virus-specific CD8 T cell response [15–17]. Strong expression of Th2 cytokines such as IL-13 are commonly implicated in disease pathogenesis [18]. However, recent results from humans and mice have suggested that IL-17-dependent immune activation may also be a critical factor in the response to RSV infection [19–23].

IL-17 is a pro-inflammatory cytokine that is primarily produced by activated γδ T cells and CD4^+^ T cells, termed Th17 cells [24]. The main effector cytokines associated with Th17 immunity are IL-17A, (IL-17), IL-17F, IL-21 and IL-22. IL-17, like IFNγ, is a “master regulator” of downstream cytokine and chemokine responses. Expression of IL-17 stimulates stromal and epithelial cells to produce chemokines such as CXCL1, CCL20, IL-6, and most importantly, IL-8, leading to recruitment and activation of neutrophils and monocytes [25]. While critical for protection from several types of extracellular bacterial and fungal infections, the role of IL-17 and Th17 cells in immunity against intracellular pathogens such as RSV remains less clear. Faber *et al*. reported significantly lower levels of IL-17 in the bronchoalveolar lavage (BAL) of severely ill, pediatric RSV patients compared to less severe cases [19]. Similarly, a study by Larranaga *et al*. revealed higher plasma levels of IL-17 in infants with moderate RSV infection compared to those with severe disease [26]. In contrast, Mukherjee *et al*. observed increased levels of IL-17 in tracheal aspirates from severely ill infants compared to healthy controls [21]. Mice deficient in STAT1 [27] or CCR7 [28] display increased pathology in the lungs that is attributed to increased production of IL-17; and animals deficient in IL-17 display significantly reduced mucus production and increased CD8 T cell responses in the lungs during primary RSV infection [21]. The role of IL-17 in RSV-associated allergic sensitization appears similarly complex, with reports that this cytokine both prevents [22] and promotes [21] airway reactivity.

Here, we report for the first time that calves infected with BRSV express increased levels of IL-17 in the lungs, and develop virus-specific IL-17 producing lymphocytes in the blood by day 7 after infection. Similarly, calves vaccinated with a live-attenuated strain of BRSV mount virus-specific IL-17 responses in the blood. Both CD4 and γδ T lymphocytes have the capacity to secrete IL-17 in response to viral infection. Using an *in vitro* co-infection model of BRSV and *M*. *haemolytica* to model BRDC, we further demonstrate that BRSV-induced expression of IL-17 is significantly exacerbated in the presence of secondary bacterial exposure to *M*. *haemolytica*, suggesting a mechanism that may be contributing to neutrophilia in the lungs of calves with BRDC. Interestingly, this exacerbated IL-17 production appears primarily mediated by γδ T cells, as depletion of these cells from culture ablates the effect. Together, our results demonstrate that calves, like humans and mice, mount a significant IL-17 response during acute BRSV infection and suggest a possible role for γδ T lymphocytes in contributing to BRDC-associated immunopathology in the calf.

## Materials and Methods

### Animals

#### BRSV infected animals

Lung samples and peripheral blood samples were collected over 3 experiments from a total of 16, 4–6 week old Holstein calves. Eight calves were infected with BRSV Strain 375 via aerosol inoculation as previously described [29, 30]. Eight uninfected, age-matched calves served as negative controls. All animals were housed in a climate-controlled, biosafety level-2 facility at the National Animal Disease Center in Ames, IA. Peripheral blood was collected via the jugular vein on day 0 prior to infection, and on day 3 and 7 post infection. Calves were monitored daily following experimental infection. Clinical signs were evaluated including rectal temperature, evidence of ocular or nasal discharge, cough, dyspnea, and appetite. The animal care protocol included provisions for a humane endpoint as determined by the discretion of the attending clinical veterinarian. No animals died or required euthanasia prior to the experimental endpoint. Methods to minimize pain and distress included the avoidance of prolonged restraint and the inclusion of euthanasia as an intervention strategy. Fundamental to the relief of pain and distress is the ability to recognize clinical signs of RSV in calves. The investigator provides assurance that any unrelieved pain and distress occurred only for the minimum time necessary to achieve study objectives and was based on sound scientific rationale. Calves were euthanized by barbiturate overdose on day 7-post infection. Lungs were removed and samples from representative gross lesion and nonlesion tissue collected from each calf and stored in RNA*later* (Invitrogen, Life Technologies).

#### *M*. *haemolytica* infected animals

Lung samples were collected from a total of 8, 6-month old castrated male Holstein calves. All calves were housed at the Large Animal Research Facility at Kansas State University. Four calves were infected via endoscope-guided challenge with 3x10^8^ CFU/mL of a field strain of *M*. *haemolytica* serotype A1 previously isolated from the lung of a bovine with BRDC [31, 32]. Protocols for growing and administering the challenge inoculum have been previously described [31, 32]. Four calves served as uninfected controls and received only PBS via endoscope-guided administration. Calves were monitored twice daily following experimental infection. Clinical signs were evaluated including rectal temperature, evidence of ocular or nasal discharge, cough, dyspnea, and appetite. The animal care protocol included provisions for a humane endpoint as determined by the discretion of the attending clinical veterinarian. Methods to minimize pain and distress included the avoidance of prolonged restraint and the inclusion of euthanasia as an intervention strategy. No animals died or required euthanasia prior to the experimental endpoint. Calves were euthanized by captive bolt on day 3-post infection. Lungs were removed and samples from representative gross lesion and non-lesion tissue collected from each calf and stored in RNAlater.

#### BRSV vaccinated animals

PBMC were collected from a total of 16 female adult Holstein cows housed at the dairy facility at the National Animal Disease Center in Ames, IA (n = 8) or the Kansas State Dairy in Manhattan, KS (n = 8). All cows were on an annual vaccination program and received a multivalent commercial vaccine containing live attenuated BRSV (BoviShield Gold FP5) within 4–12 weeks of inclusion in the study. No experimental endpoint was established for these animals, as they were healthy and were not experimentally infected. All provisions to minimize pain and distress were used including phlebotomy by experienced handlers and avoidance of prolonged restraint. All animals tested positive for antibodies to BRSV (titer of 1:32–1:128), and positive for BRSV-specific CD4 T cell responses prior to enrollment into the study. Six cows that were not on the annual vaccination program due to inclusion in another study were used as nonvaccinated controls. Control cows were also housed at the Kansas State University Dairy in Manhattan, KS. These animals had a BRSV titer of 1:8–1:64, but had no detectable BRSV-specific CD4 T cell responses as measured by T cell proliferation assays.

All animal studies were conducted in strict accordance with federal and institutional guidelines and were approved by the National Animal Disease Center Institutional Animal Care and Use Committee or the Kansas State University Institutional Animal Care and Use Committee, as appropriate.

### Peripheral blood collection and mononuclear cell preparation

Mononuclear cells were isolated by density centrifugation of peripheral blood collected from the jugular vein into 2x acid citrate dextrose. Contaminating red blood cells were removed using hypotonic lysis. Cells were washed and resuspended in complete RPMI (cRPMI) as previously described [30].

CD4 T cells and γδ T cells were enriched from PBMC using Magnetic Activated Cell Sorting (MACS) as previously published [33]. Briefly, PBMC were labeled with 10 μg/mL mouse anti-bovine CD4 (clone ILA11A) or 10 μg/mL mouse anti-bovine γδ T cell receptor (Clone GB21A), both from Washington State Monoclonal Antibody Center (Pullman, WA). Cells were then labeled with anti-mouse IgG2A+B magnetic beads (Miltenyi Biotech) and purified by positive sorting per manufacturer’s instructions.

WC1 expressing subsets of γδ T cells were isolated by FACS sorting as previously described [30, 33]. PBMC were labeled with mouse anti-bovine WC1.1 (Clone BAG25A), mouse anti-bovine WC1.2 (Clone CACTB32A) and mouse anti-bovine γδ T cell receptor (Clone GB21A), all from Washington State Monoclonal Antibody Center (Pullman, WA). Cells were then labeled with anti-mouse IgG1-AF488, anti-mouse IgM-PE and anti-mouse IgG2b-AF647 secondary antibodies (Invitrogen, Life Technologies). Cells were sort purified based upon their expression of the γδ T cell receptor and WC1.1, WC1.2 or lack of WC1 expression. Subsets were sorted to > 90% purity using a BD FACS Aria Cell Sorting System (BD Biosciences, San Jose, CA).

Monocytes were isolated by plastic adherence as previously described [30, 33]. Briefly, PBMC were suspended in cRPMI and allowed to adhere to plastic Petri dishes for 2 hr at 37°C. Non-adherent cells were removed by washing with warm cRPMI. Adherent cells were collected by washing with ice-cold PBS and gentle scraping.

### *In vitro* cell stimulation

PBMC or MACS purified T lymphocytes were plated at a concentration of 4x10^6^ cells/mL in cRPMI (100 μl/well) in sterile, round-bottom, 96-well tissue-culture-treated plates (BD Biosciences). For experiments requiring antigen-presenting cells (APC), monocytes were plated at a ratio of 1:5 with purified CD4 or γδ T cells. In some experiments, cells were labeled with Cell Trace Violet per manufacturer’s recommendations (Invitrogen, Life Technologies) prior to being placed in culture. For *in vitro* re-stimulation with BRSV, cells were incubated at a 0.1 multiplicity of infection (MOI) with BRSV Strain 375 for 90 min at 37°, washed once and resuspended in cRPMI for the remaining incubation period. For experiments using heat-killed BRSV, virus stock was inactivated for 30 minutes at 56°C, then 50 μl added to the cultures (final volume of 200 μl/well).

For *in vitro* BRDC experiments, cells were infected with BRSV Strain 375 as above. Four hours later, *M*. *haemolytica* strain D153, serotype A1 or *Pastuerella multocida* strain P1062, serotype A3 was suspended in antibiotic-free RPMI and added to the cell cultures at ~0.1 MOI as determined by spectrophotometric growth curves. After 3–4 hours, cells were resuspended in antibiotic-containing cRPMI to prevent bacterial overgrowth. Importantly, we performed dose titrations to determine the optimal concentration of *M*. *haemolytica* to add to our cultures. The addition of higher concentrations of *M*. *haemolytica* resulted in significant cell death, likely a result of the action of leukotoxin [34–36], while an MOI of 0.5 or less did not induce significantly increased cell death over uninfected cultures (not shown). Heat-inactivated *M*. *haemolytica* D153 was prepared by suspending bacteria in antibiotic-free RPMI at approximately 0.1 MOI, then boiling for 15 minutes. Inactivated *M*. *haemolytica* was then added to PBMC cultures for 3–4 hours, followed by centrifugation and a media change to antibiotic-containing cRPMI as above.

At the indicated times (24 hours or 6 days of culture), PBMC, CD4 or γδ T cells were pelleted by centrifugation and cell culture supernatants were collected and stored at −80°C. Cell pellets were then lysed with Buffer RLT (Qiagen, Valencia, CA) containing 2-mercaptoethanol and stored at −80°C.

### Real-time PCR

RNA was isolated from lung tissue samples using Trizol Reagent (Invitrogen, Life Technologies) according to manufacturer’s instructions. The RNA concentration in each sample was determined using a NanoDrop 2000 spectrophotometer (Thermo Scientific, Wilmington, DE). RNA (300 ng per sample) was DNase-treated and cDNA synthesized using random primers and Superscript III Reverse Transcriptase according to the manufacturer's instructions (Invitrogen, Life Technologies).

For PBMC, purified T cell populations and bovine turbinates (BT), RNA was extracted using the RNeasy Mini RNA Isolation kit (Qiagen) according to manufacturer's instructions. Contaminating genomic DNA was removed using the RNase-Free DNase digestion set (Qiagen) as per manufacturer's instructions. Total eluted RNA was reverse transcribed into cDNA using Superscript III Reverse Transcriptase and Random Primers following the manufacturer's instructions.

Quantitative real-time PCR (qPCR) was performed using Power SYBR Green PCR Master Mix (Applied Biosystems, Carlsbad, CA). Forward and reverse primers used in the study are listed in Table 1. Reactions were performed on a 7300 Real-Time PCR System (Applied Biosystems, Life Technologies, Carlsbad, CA) or an Mx3005P qPCR System (Agilent Technologies, Santa Clara, CA). The following amplification conditions were used: 2 min at 50°, 10 min at 95°, 40 cycles of 15 seconds at 95° and 1 min at 60°, and a dissociation step (15 seconds at 95°, 1 min at 60°, 15 seconds at 95°, 15 seconds at 60°). Relative gene expression was determined using the 2^-ΔΔCt^ method with RPS9 as the reference housekeeping gene.

**Table 1:** Primers used for qPCR.

Gene (alternate name)	Accession number	Strand	Sequence (5’-3’)	Reference
IL17	NM_001008412	Forward	CACAGCATGTGAGGGTCAAAC	[37]
		Reverse	GGTGGAGCGCTTGTGATAAT	
IL-21	NM_198832	Forward	GTGGCCCATAAGTCAAGCTC	[38]
		Reverse	CGCTCACAGTGTCTCTTTAC	
IL-22	NM_001098379	Forward	GACTGTGGAGTTTGGCTCCCCCTTTTC	[38]
		Reverse	GAGATTAAAGTCATTGGAGAACTGAAC	
IL-8	X78306	Forward	AAGCTGGCTGTTGCTCTC	[29]
		Reverse	GGCATCAGAAGTTCTGTACTC	
MUC5AC	BN001491	Forward	CAGACCCTCCACCTTCTTCA	[39]
		Reverse	GGTCCTCGAAGCTGTTCTTG	
MUC5B	BN0011492	Forward	TCTACCTGACCGTGGAGACC	[39]
		Reverse	GTTGATGATGCTGCACTGCT	
RPS9	NM_001101152.1	Forward	CGCCTCGACCAAGAGCTGAAG	[29]
		Reverse	CCTCCAGACCTCACGTTTGTTCC	

### IL-17 ELISA

The Bovine IL-17A VetSet ELISA Development kit was purchased from Kingfisher Biotech, Inc. (St. Paul, MN). ELISAs were performed using cell culture supernatants according to manufacturer’s instructions.

### Stimulation of BT

BT cells were cultured in cMEM, plated in 96-well, flat-bottom tissue-culture-treated plates and grown to confluence overnight at 37°C. Cell culture supernatants from day 6 stimulated PBMC, CD4 or γδ T cell culture supernatants were diluted 1:2 with cMEM, and then added to the BT cells. Cells were incubated overnight, then lysed with Buffer RLT (Qiagen, Valencia, CA) containing 2-mercaptoethanol and stored at −80°C until RNA extraction.

### Statistical Analysis

ΔΔCt values were used in the statistical analysis of relative gene expression. ΔΔCt values were transformed (2^-ΔΔCt^) and are shown as expression relative to uninfected or unstimulated control samples, as appropriate. [40]

Statistical analyses were performed using Prism v6.0f software (Graphpad Software, Inc.) Statistical comparisons were performed using a two-tailed Student’s *t*-test when comparing control to infected or stimulated samples; or a one-way ANOVA with Bonferri post-test analysis, when comparing multiple treatment conditions (*in vitro* BRDC studies).

## Results

### IL-17 response in the lungs and peripheral blood of calves infected with bovine RSV

**Fig 1:**
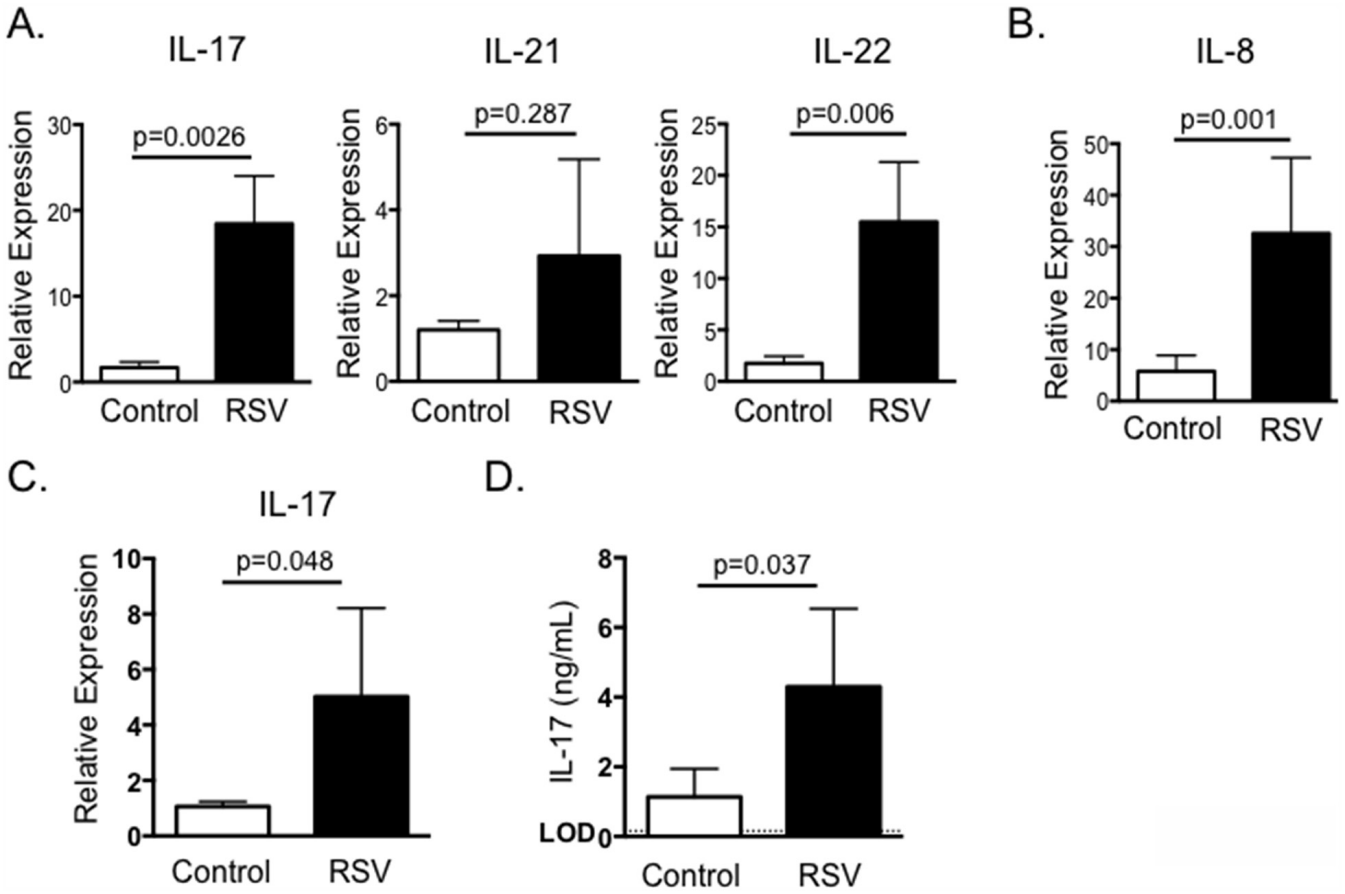
IL-17 and Th17 responses in the lungs and peripheral blood of calves infected with BRSV. Calves (n = 8) were infected via aerosol inoculation with BRSV Strain 375 (RSV) as described in Materials and Methods. Control calves remained uninfected (n = 8). On day 7 post-infection, the animals were sacrificed and the lungs analyzed by qPCR for expression of IL-17, IL-21 and IL-22 (A) and IL-8 (B). In separate experiments, PBMC were isolated from control (n = 8) and BRSV infected calves (n = 8) on day 7-post infection. PBMC were stimulated with BRSV for 24 hours and then analyzed for expression of IL-17 by qPCR (C); or for 6 days and then cell culture supernatants were analyzed by ELISA for expression of IL-17 (D). For qPCR analysis, results were normalized to the housekeeping gene RPS-9, and expressed relative to samples from uninfected control calves. Results were pooled from a total of three independent experiments. Data represent means ± SEM.

IL-17 expression is increased in mice and humans with acute RSV infection [20–23, 41], but has not been studied in calves with acute BRSV infection. To this end, neonatal Holstein calves were infected with BRSV strain 375 via aerosol inoculation. On day 7-post infection, lung samples were analyzed by qPCR for expression of IL-17 and the Th17-associated cytokines IL-21 and IL-22. As seen in Fig 1, BRSV infection resulted in significantly increased expression of IL-17 and IL-22 in the lungs of infected calves (Fig 1A). IL-21 expression was not significantly increased in pneumonic lung at this time.

IL-17 promotes increased production of the pro-inflammatory chemokine, IL-8. IL-8 is increased in humans with RSV [42, 43] and we have previously demonstrated that IL-8 is upregulated in the lungs of calves with BRSV [29]. In agreement with these previous reports, IL-8 expression was significantly increased in the lung lesions of calves infected with BRSV compared to lungs from uninfected control calves (Fig 1B).

To determine if BRSV infection induced the development of systemic Th17 responses, peripheral blood was collected from control and BRSV-infected calves on day 7-post infection. PBMC were restimulated with heat-killed BRSV antigen for 24 hours (Fig 1C) and examined by qPCR for expression of IL-17. PBMC from calves with acute BRSV infection expressed significantly increased levels of IL-17 in recall response to BRSV infection. Importantly, significant IL-17 expression was not observed in PBMC collected from the calves on day 0 or 3-post infection (data not shown). PBMC were also stimulated with heat-killed BRSV antigen for 6 days and culture supernatants analyzed by ELISA. In agreement with the qPCR results, PBMC from infected, but not control calves secreted significantly increased concentrations of IL-17 in recall response to BRSV antigen (Fig 1D).

### IL-17 response in the peripheral blood of calves vaccinated with live-attenuated BRSV

**Fig 2:**
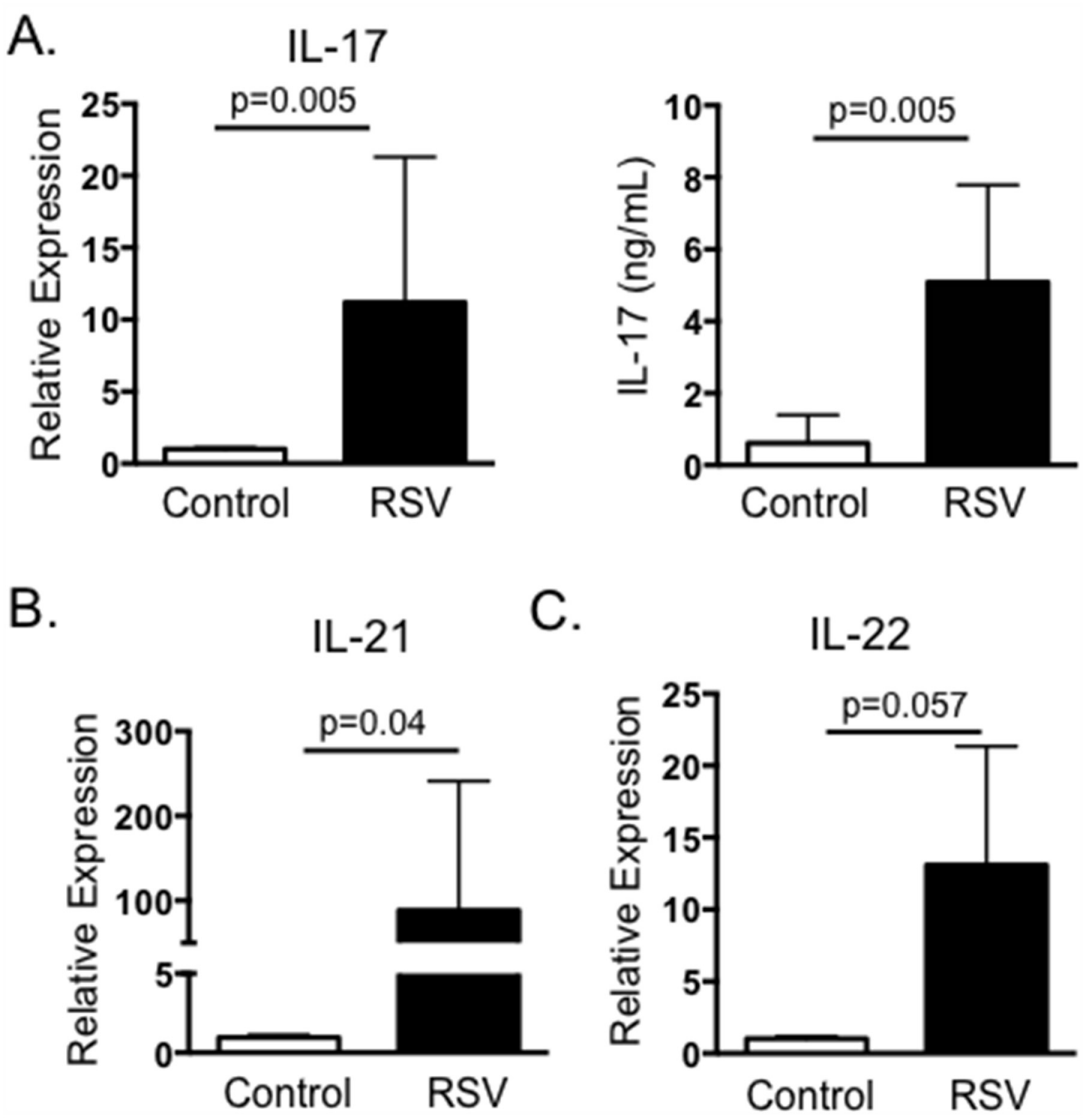
IL-17 and Th17 responses from BRSV vaccinated cattle. Peripheral blood was collected from cows receiving annual vaccinations with a multivalent vaccine containing live-attenuated BRSV (n = 8), or from control cows that were not included in the vaccination program due to inclusion in another study (n = 6). PMBC were isolated and stimulated with BRSV for 24 hours or 6 days, as in Fig 1. RNA was isolated from the cells and analyzed by qPCR for expression of IL-17 (A, left panel), IL-21 (B) and IL-22 (C). Cell culture supernatants were also analyzed by ELISA for IL-17 (A, right panel). For qPCR analysis, results were normalized to the housekeeping gene RPS-9, and expressed relative to unstimulated control samples. Results were pooled from two independent experiments. Data represent means ± SEM.

We next chose to determine if BRSV vaccination also induces the development of virus-specific IL-17 responses. PBMC were collected from adult Holstein cows within 4–12 weeks of receiving an annual booster of live, attenuated BRSV vaccine. Peripheral blood was also collected from control cows that were not included in the annual vaccination program due to inclusion in another study. PBMC from both groups of animals were stimulated with heat-killed BRSV strain 375 and assessed by qPCR 24 hours later for expression of IL-17. In agreement with our results from calves with acute BRSV infection, cells from BRSV vaccinated animals produced virus-specific IL-17 as measured by qPCR (Fig 2A) and by ELISA (Fig 2B). Similar to our results from BRSV infected calves, PBMC from vaccinated cows also expressed increased levels of IL-21 and IL-22 in recall responses to BRSV antigen (Fig 2).

**Fig 3:**
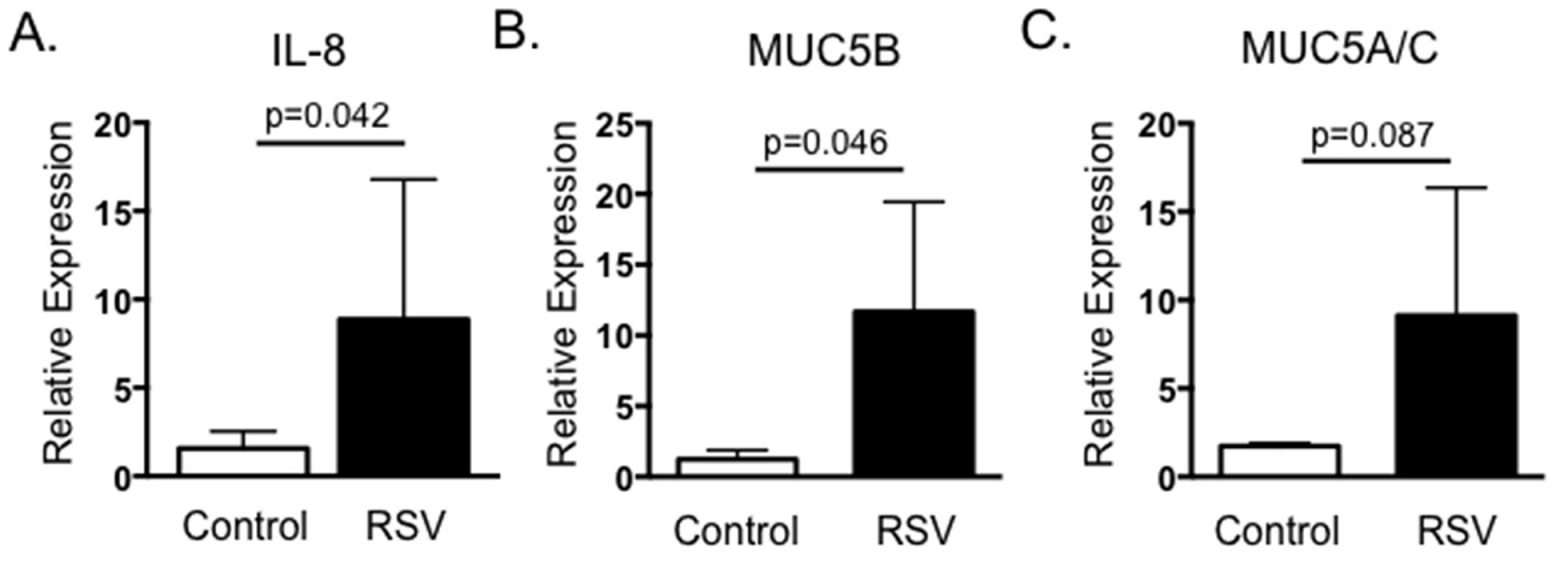
Cell culture supernatants from BRSV stimulated PBMC induce expression of IL-8, MUC5B and MUC5AC by BT cells. PBMC from control (nonvaccinated) and BRSV vaccinated cows were isolated and stimulated with heat-killed BRSV for 6 days as in Fig 2. Cell culture supernatants were then diluted 1:1 in cMEM and added to confluent BT cells in 96 well plates for 24 hours. RNA was the isolated from the BT and analyzed by qPCR for expression of IL-8 (A), MUC5B (B) and MUC5AC (C). For qPCR analysis, results were normalized to the housekeeping gene RPS-9, and expressed relative to unstimulated control samples. Results were pooled from two independent experiments. Data represent means ± SEM.

Severe RSV infection in humans is associated with increased neutrophil infiltration and increased mucus production. IL-17 reportedly increases expression of IL-8, a critical neutrophil chemoattractant, and the genes encoding mucins, MUC5AC and MUC5B [24]. To confirm that IL-17 and the Th17 associated cytokines induced by BRSV displayed similar activity in the bovine, we treated BT cells for 24 hours with supernatants collected from BRSV antigen-stimulated cultures. BT cells were then analyzed by qPCR for expression of IL-8 (Fig 3A), MUC5AC (Fig 3B) and MUC5B (Fig 3C). As seen in Fig 3, cell culture supernatants from non-vaccinated cows that were stimulated with BRSV induced only minor expression of IL-8, MUC5B and MUC5AC by BT, while stimulated supernatants from vaccinated calves induced increased expression of all three target genes by BT cells.

### CD4 and γδ T cells produce IL-17 in response to BRSV

**Fig 4:**
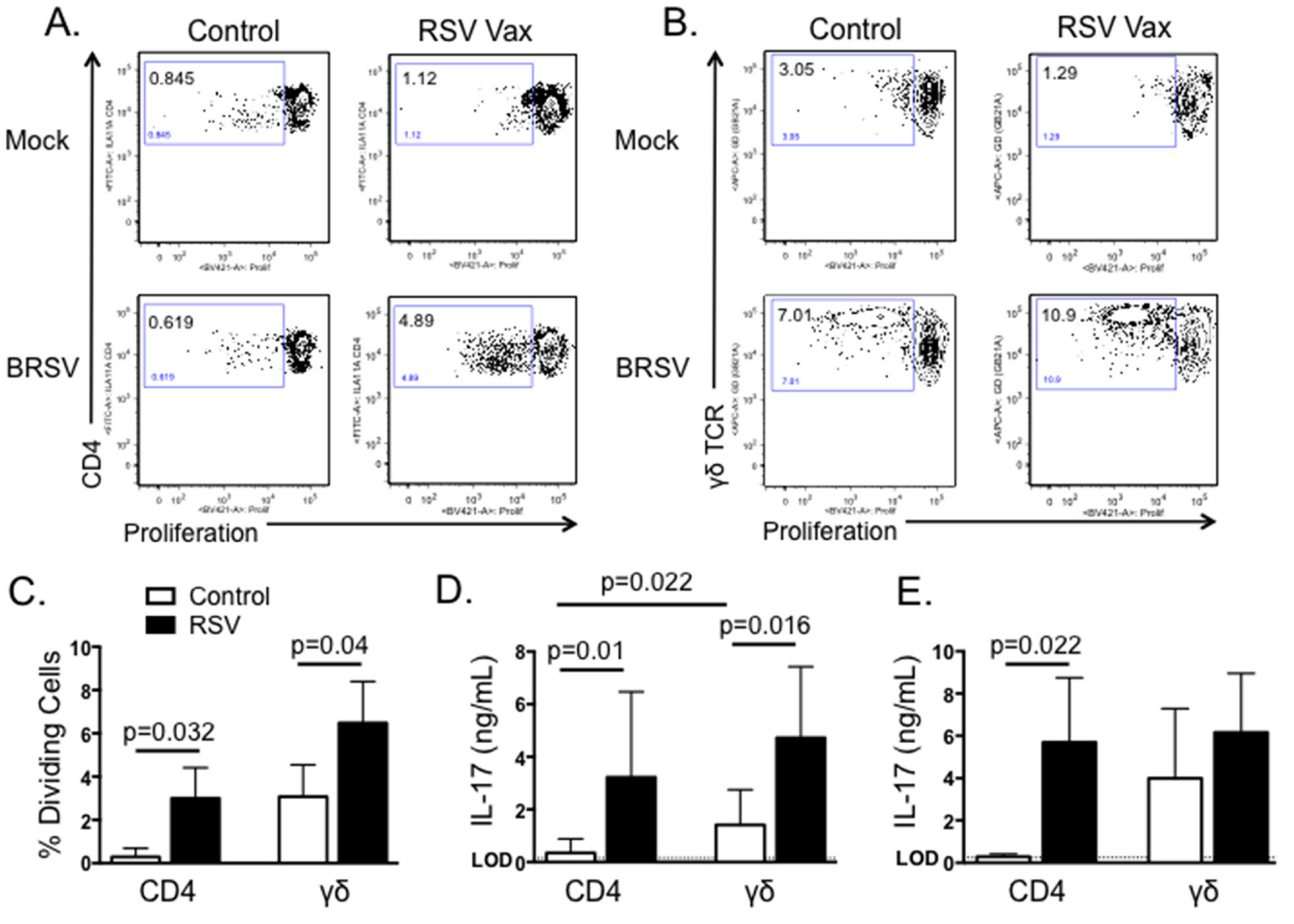
Both CD4 T cells and γδ T cells produce IL-17 in response to BRSV. PBMC were isolated from control or BRSV vaccinated cows and labeled with Cell Trace Violet. Cells were then cultured for 6 days with BRSV. On day 6, CD4 T cells (A) and γδ T cells (B) were analyzed for virus-specific proliferation as measured by Cell Trace Violet dilution. Representative flow plots are shown in A and B. Aggregate results are shown in C. (D) CD4 T cells and γδ T cells from BRSV vaccinated or nonvaccinated animals were isolated by MACS and cultured in the presence of autologous APC ± BRSV. After 6 days, cell culture supernatants were analyzed by ELISA for IL-17. (E) CD4 T cells and γδ T cells were MACS purified from peripheral blood of calves infected or not with BRSV strain 375 for 7 days. Purified cells were cultured in the presence of autologous APC ± BRSV. After 6 days, cell culture supernatants were analyzed by ELISA for IL-17. For A-C, background levels of proliferation were subtracted and results are presented as change over mock. Results are pooled from two independent experiments. Data represent means ± SEM.

Both CD4 T cells and γδ T cells have the capacity to produce IL-17 in response to RSV infection in humans and mice [24]. Therefore, we next chose to determine which T cell subsets from cattle produce IL-17 in recall response to BRSV stimulation. PBMC from BRSV vaccinated cattle were labeled with CellTrace Violet, then cultured for 6 days in the presence or absence of heat-killed BRSV. CD4 T cells and γδ T cells were then analyzed by flow cytometry for BRSV-specific proliferation as measured by CellTrace Violet dilution. As seen in Fig 4A–4C, both CD4 T cells and γδ T cells from vaccinated cows proliferate in specific response to stimulation with BRSV antigen. Not surprisingly, we observed higher levels of non-specific proliferation by γδ T cells from control, non-vaccinated cows; however, BRSV-induced proliferation was still significantly increased above background levels (Fig 4C). Unfortunately, an antibody is not available for intracellular cytokine staining of bovine IL-17. Therefore, CD4 T cells and γδ T cells were purified by MACS and cultured with autologous APC in the presence or absence of BRSV antigen for 6 days. Cell culture supernatants were then analyzed by ELISA to determine concentrations of IL-17. As seen in Fig 4D, both CD4 T cells and γδ T cells secrete IL-17 in specific response to BRSV antigen. γδ T cell production of non-specific IL-17 was increased compared to CD4 T cells, but stimulation with BRSV antigen resulted in a significant increase in virus-specific IL-17 production by purified γδ T cells. Importantly, we observed similar IL-17 production when analyzing CD4 and γδ T cells isolated from peripheral blood of BRSV infected calves (Fig 4E).

**Fig 5:**
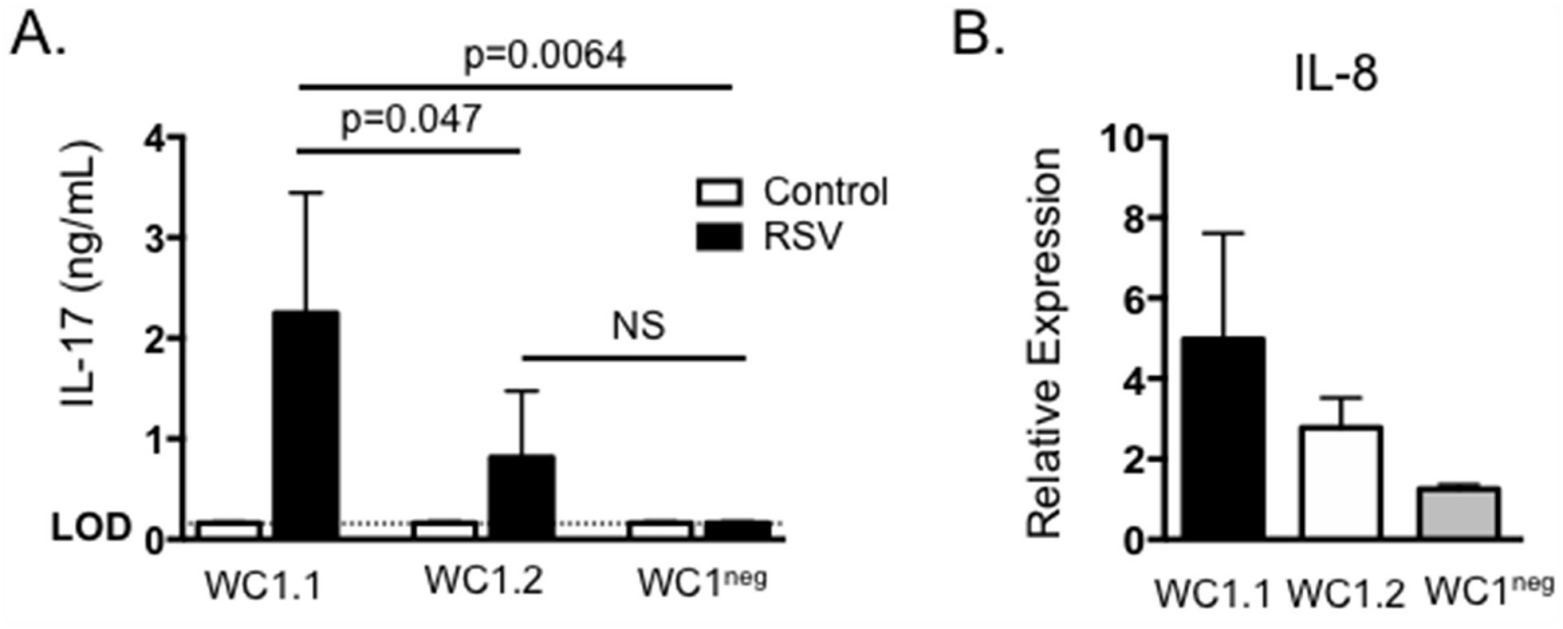
WC1.1^+^ γδ T cells produce IL-17 in response to BRSV. γδ T cells were purified from the peripheral blood of BRSV vaccinated or control animals by FACS based upon their expression of the γδ T cell receptor and either expression of WC1.1, WC1.2 or lack of WC1. Cells were cultured in the presence of autologous APC ± BRSV for 6 days. Cell culture supernatants were then analyzed by ELISA for IL-17. (B) Cell culture supernatants from (A) were also diluted 1:1 and added to BT cells for 24 hours. After 24 hours, BT were analyzed by qPCR for expression of IL-8. For qPCR analysis, results were normalized to the housekeeping gene RPS-9, and expressed relative to unstimulated control samples. Results are pooled from two independent experiments. Data represent means ± SEM.

In cattle, γδ T cells are phenotypically and functionally divided into subsets based upon their expression of WC1. We have previously demonstrated that WC1^neg^, WC1.1^+^ and WC1.2^+^ γδ T cell subsets respond to BRSV infection, and the subsets differ in their response to virus with regards to inflammatory chemokine expression, IFNγ production and production of regulatory cytokines such as IL-10 [30]. We next chose to determine the contribution of individual WC1-expressing subsets in production of IL-17 during the response to BRSV. γδ T cells were FACS purified into subsets based upon their expression of the γδ T cell receptor and WC1.1, WC1.2 or lack of WC1 as previously described [30, 33]. Cells were cultured as in Fig 4 and then supernatants were assessed for concentrations of IL-17 by ELISA. As seen in Fig 5A, WC1.1^+^ γδ T cells secreted significant levels of IL-17 in response to BRSV antigen, while neither WC1.2^+^ nor WC1^neg^ subsets produced IL-17, suggesting the WC1.1 subset may be the primary γδ T cell-derived source of IL-17 during BRSV infection in the calf. Stimulation of BT cells with culture supernatants from all three subsets of purified γδ T cell subsets resulted in an increase in IL-8 expression (Fig 5B).

### IL-17 expression in the lungs during *M*. *haemolytica* infection

**Fig 6:**
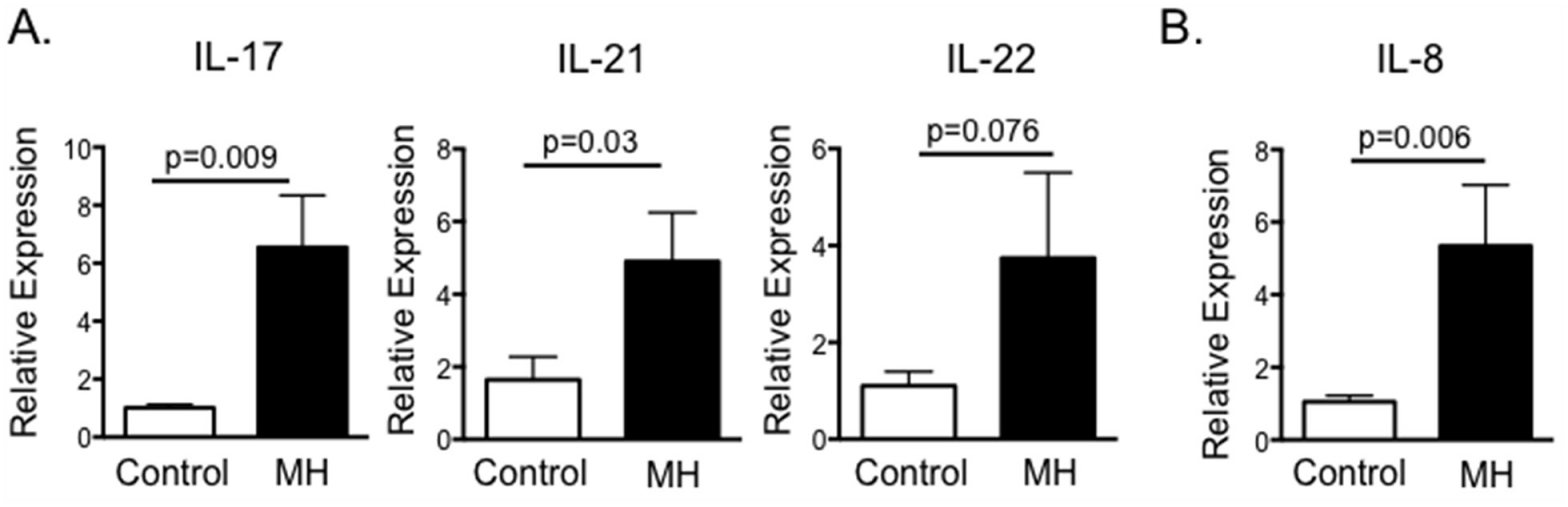
*M*. *haemolytica* infection induces IL-17 and Th17 cytokine production. Calves (n = 4) were infected with a field strain of *M*. *haemolytica* via endoscope-guided inoculation as described in Materials and Methods. Control calves were sham inoculated (n = 4). On day 3 after infection, animals were sacrificed and their lungs analyzed by qPCR for expression of IL-17, IL-21 and IL-22 (A); and for expression of IL-8 (B). Results were normalized to the housekeeping gene RPS-9, and expressed relative to samples from uninfected control calves. Results are pooled from one experiment. Data represent means ± SEM.

*M*. *haemolytica* is the predominant bacterial isolate recovered from cases of bovine pneumonia and the leading cause of direct economic loss from BRDC in the United States [44]. IL-8 is upregulated in the lungs of *M*. *haemolytica* infected cattle [45, 46], and significant numbers of neutrophils are recruited to the lungs following infection, resulting in the exacerbated inflammation and tissue damage typical of BRDC [8, 34]. We next determined if IL-17 is contributing to this increase in IL-8 production and neutrophil recruitment during *M*. *haemolytica* infection. To this end, cattle were infected with *M*. *haemolytica* via endoscope-guided inoculation. On day 3 after inoculation, animals were sacrificed and their lungs examined by qPCR for expression of IL-17. As seen in Fig 6A, *M*. *haemolytica* infection results in increased expression of IL-17, IL-22 and IL-23 in the lungs by day 3 after infection. In agreement with previous studies [45, 46], we also observed significantly increased expression of IL-8 in lung tissue from *M*. *haemolytica* infected calves compared to controls (Fig 6B).

### *In vitro* infection with BRSV and *M*. *haemolytica* induces exacerbated production of IL-17

**Fig 7:**
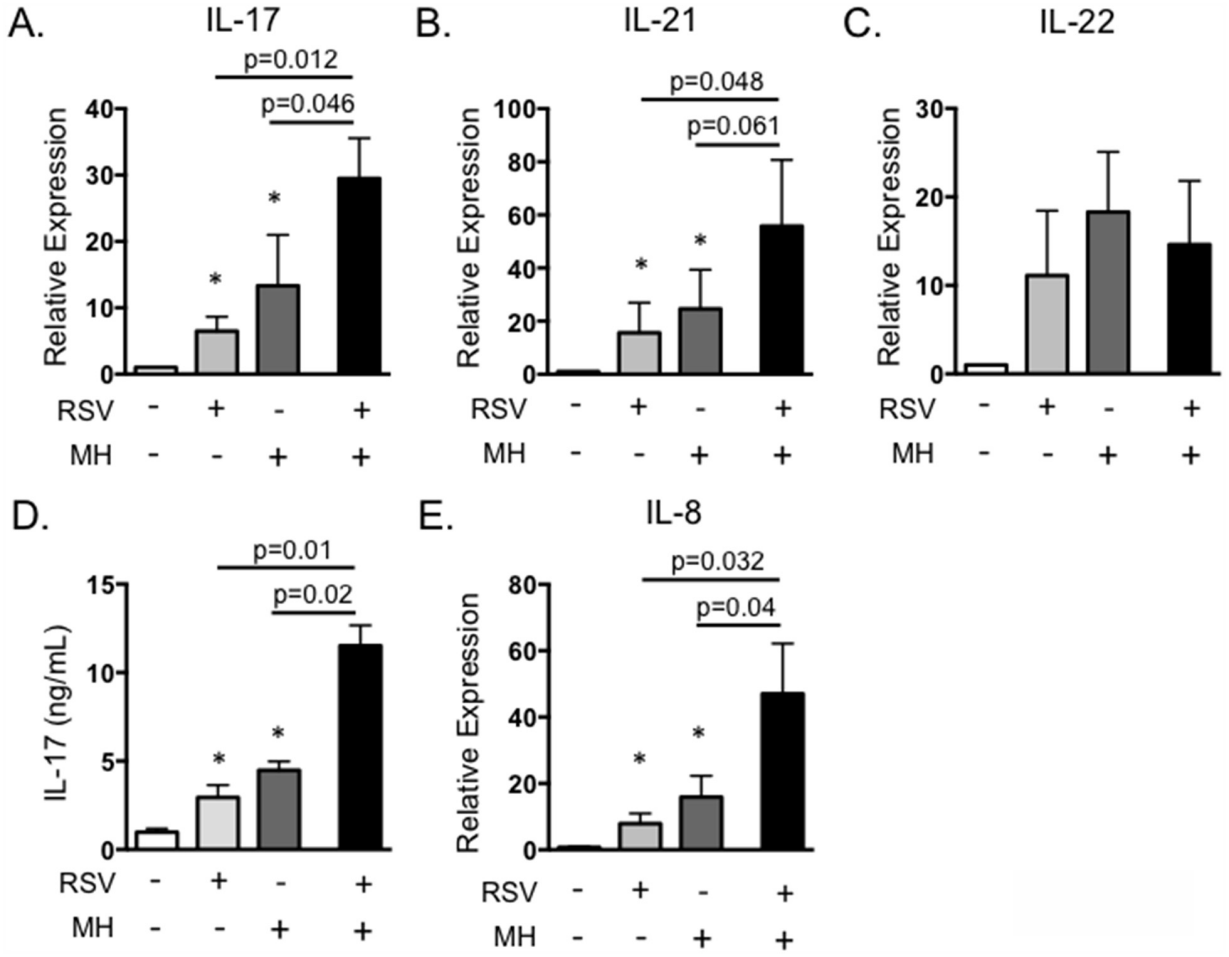
*In vitro* infection with BRSV and *M*. *haemolytica* results in exacerbated IL-17 production. PBMC from BRSV vaccinated cows were stimulated with BRSV for 6 hours, then cultured with 0.1 MOI *M*. *haemolytica* in antibiotic free media for 4 hours. After four hours, antibiotics were added, and then incubations were continued for 18 hours (A-C) or 6 days (D and E). Control cultures were included that received neither pathogen, BRSV alone or *M*. *haemolytica* alone. (A-C) Cells were analyzed by qPCR for expression of IL-17 (A), IL-21 (B) and IL-22 (C). Cell culture supernatants from day 6 were analyzed by ELISA for IL-17 (D) and were diluted 1:1 and added to BT for 24 hours (E). BT were analyzed by qPCR for expression of IL-8 after overnight stimulation as in Fig 3. Results in A-C are pooled from three independent experiments with n = 12. Results in D and E are from two experiments with n = 8. Data represent means ± SEM.

**Fig 8:**
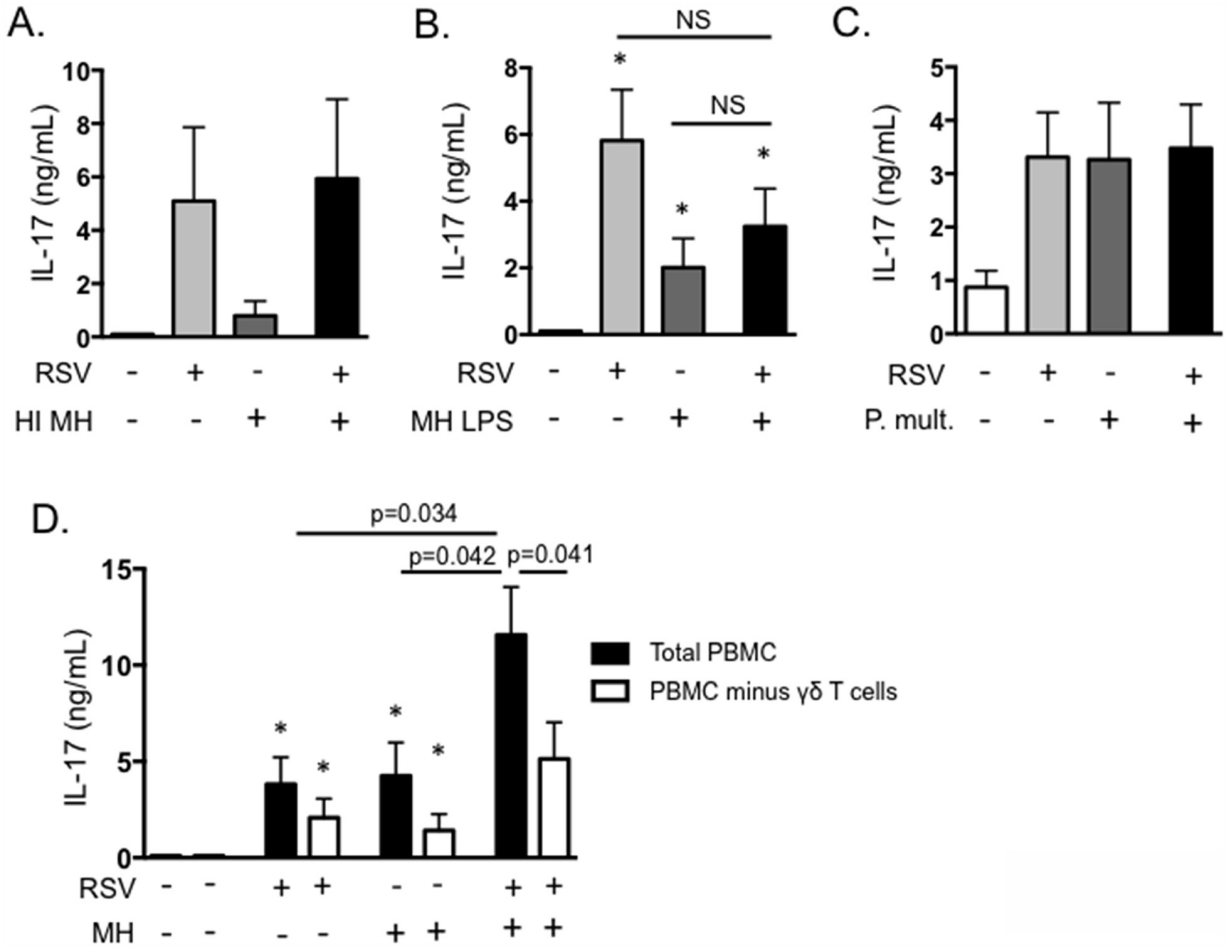
γδ T cells are the primary source of exacerbated IL-17 production in an *in vitro* model of BRDC. (A and B) PBMC from BRSV vaccinated cows were stimulated with BRSV for 6 hours, then with 0.1 MOI of heat-inactivated *M*. *haemolytica* (A) or 1 μg/mL purified LPS from *M*. *haemolytica* (B). Control cells remained unstimulated, were stimulated with only BRSV or were stimulated with only heat-inactivated *M*. *haemolytica* LPS. After six days, cell culture supernatants were analyzed by ELISA for IL-17. (C) PBMC from BRSV vaccinated cows were stimulated with BRSV for 6 hours, then cultured with 0.1 MOI *P*. *multocida* in antibiotic free media for 4 hours. After four hours, antibiotics were added, and then incubations were continued for 6 days. Control cultures were included that received neither pathogen, BRSV alone or *P*. *multocida* alone. Cell culture supernatants were analyzed by ELISA for IL-17. (D) PBMC were depleted of γδ T cells using MACS. Total PBMC and γδ T cell-depleted PBMC were then cultured in the presence of BRSV and *M*. *haemolytica* as above. After 6 days, cell culture supernatants were analyzed by ELISA. Results in A-C are from one experiment with n = 4. Results in D are pooled from 2 independent experiments with n = 8. Data represent means ± SEM.

Primary BRSV infection is a significant factor promoting the development of secondary bacterial pneumonia caused by pathogens such as *M*. *haemolytica*. We are interested in determining mechanisms of immunity that predispose an animal to the development of BRDC. IL-17 plays a critical role in neutrophil recruitment and inflammation, and neutrophilia in the lungs is a trait characteristic of severe pasteruellosis in calves with BRDC [45]. Therefore, we chose to determine the effect of secondary bacterial exposure on IL-17 expression and its possible role in the development of exacerbated bacterial pneumonia. To this end, we developed an *in vitro* model of BRDC. PBMC were infected with BRSV, followed by exposure to live *M*. *haemolytica*, mimicking a situation that may occur in the lungs, where T lymphocytes recruited to control BRSV infection are exposed to *M*. *haemolytica* during secondary bacterial infection. In Fig 7, PBMC were infected with 0.1 MOI of BRSV strain 375 for 6 hours, then *M*. *haemolytica* strain D153 was added to the cultures at an MOI of 0.1. Cells and bacteria were incubated for 4 hours in antibiotic free media, then antibiotics were added to control possible bacterial overgrowth. Cells were stimulated for 18 hours and then analyzed by rtPCR, and were also cultured for 6 days and supernatants measured by ELISA. *M*. *haemolytica* produces a potent leukotoxin that can induce rapid cell death in activated bovine PBMC [34–36]. Thus, dose titration experiments were performed to determine the optimal dose of *M*. *haemolytica* to add that did not induce significantly increased cell death in our PBMC cultures. Doses of *M*. *haemolytica* above 0.5 MOI induced significantly increased cell death in our PBMC coinfection cultures (data not shown), thus we selected an MOI of 0.1. As seen in Fig 7A, cells exposed to either BRSV alone or *M*. *haemolytica* alone secreted IL-17. However, IL-17 was significantly increased in the presence of both BRSV and *M*. *haemolytica*. A similar exacerbation of IL-21 expression was also observed in the presence of both BRSV and *M*. *haemolytica*, but not following stimulation with either pathogen singly (Fig 7B). In contrast, expression of IL-22 was not increased following exposure to both BRSV and *M*. *haemolytica* (Fig 7C). ELISA for IL-17 protein in day 6 culture supernatants confirmed our qPCR results, demonstrating increased production of IL-17 in response to BRSV or *M*. *haemolytica* alone, but significantly exacerbated production in response to the pathogens together (Fig 7D). Addition of the stimulated cell supernatants to BT cells as in Fig 3, also resulted in significantly increased production of IL-8 (Fig 7E). This suggests that the increased production of IL-17 during co-infections may significantly alter inflammatory cytokine production by epithelial and stromal cells in the lungs, leading to the severe neutrophilia that is a hallmark of the BRDC phenotype (Fig 7). Interestingly, a similar result is not observed in response to heat-inactivated *M*. *haemolytica* (Fig 8A) or in response to purified *M*. *haemolytica* LPS (Fig 8B).

*Pasteruerella multocida* is also a known cause of BRDC in young calves. Like *M*. *haemolytica* infection, pathogenesis of *P*. *multocida* pneumonia includes severe neutrophilia in the lungs [9]. We next determined if co-infection with *P*. *multocida* results in a similar exacerbation of IL-17. Interestingly, although *P*. *multocida* alone promotes IL-17 production by bovine PBMC, co-culturing of PBMC with *P*. *multocida* and BRSV together did not result in the same enhanced IL-17 phenotype that was observed in response to *M*. *haemolytica* (Fig 8B).

To determine which cells were responsible for the exacerbated production of IL-17, we next conducted depletion experiments. γδ T cells were depleted from the cultures by MACS, prior to BRSV/*M*. *haemolytica* co-infection. As seen in Fig 8D, depletion of γδ T cells resulted in slight, but not significant reductions in IL-17 production in cultures exposed to BRSV or *M*. *haemolytica* individually. However, depletion of γδ T cells significantly reduced the exacerbated production of IL-17 in co-infected cultures (Fig 8D). Depletion of CD4 T cells from the cultures yielded an overall reduction in IL-17 production in both singly and co-infected cultures, but did not alter the exacerbated phenotype observed following exposure to both pathogens (not shown). Together, our results suggest that γδ T cells are a significant source of IL-17 during *in vitro* virus/bacterial coinfections, suggesting γδ T cell derived IL-17 may also be important in the pathogenesis of BRDC in the calf.

## Discussion

We demonstrate here for the first time that neonatal calves infected with BRSV mount a robust IL-17 response in the lungs and peripheral blood. Both CD4 and γδ T cells are a significant source of IL-17 in recall responses to BRSV antigen. Similarly, both CD4 T cells and γδ T cells in rodents and humans contribute to IL-17 production in the lungs during RSV infection [19–23, 27, 28]. Previous results have suggested that γδ T cells may contribute to early IL-17 production, while Th17 cells are more important later in infection. On day 7 post-infection in the calf, our results indicate that both cell subsets have the capacity for significant virus-specific IL-17 production when isolated from peripheral blood. We were unable to detect BRSV-specific IL-17 production by peripheral blood lymphocytes at earlier time points after infection, although possible IL-17 expression in the lungs at early time points was not examined. In future studies, a more detailed analysis of the kinetics of IL-17 production by both cell types in the blood and at the site of virus infection in the lungs will be valuable.

Currently, the extent of γδ T cell involvement in RSV disease in humans is poorly defined. We have previously described potent chemokine and cytokine production by bovine γδ T cells responding to *in vitro* and *in vivo* BRSV infection [30]; and we demonstrate here that γδ T cells may play a critical role in IL-17 production during RSV infection in the calf. We suggest that the bovine represents an excellent model for further delineating the role of γδ T cells in protection or immunopathology in the lungs during RSV infection. γδ T cells are conserved amongst all vertebrate species, and are found in large proportions in a number of animal species including cattle, sheep, pigs and poultry [47–50]. In young cattle, γδ T cells can comprise as high as 60% of circulating blood mononuclear cells, while the percentage in adult animals declines to around 8–18% [47, 51]. The percentage of γδ T cells in humans is much smaller, ranging from 2–5% in healthy adults [52]. However, the frequency of γδ T cells circulating in human patients with certain bacterial and protozoal infections can increase to as high as 57% of circulating CD3^+^ T cells [52]. γδ T cells in both humans and cattle are hypothesized to play an immune sentinel role, and large proportions are also found in the respiratory tract, mucosa and skin of both species [53]. With the exception of their increased abundance in the bovine immune system, literature suggests many similarities between bovine and human γδ T cells. Amongst these, the ability to process and present antigens via MHC class II [54, 55], lack of MHC restriction [53, 56], potent inflammatory chemokine and cytokine production [57], and innate recognition of conserved pathogen associated molecular patterns via pattern recognition receptors such as TLR and NLR [30, 57–61].

Studies in mice and humans have demonstrated production of IL-17 during acute infection and recovery from BRSV. However, it remains debated if this response has positive or negative consequences on the host. Some reports in children have suggested increased levels of IL-17 in the BAL and plasma correlate with less severe RSV disease [19, 26]; while others have reported the opposite [21]. In mice, it appears that IL-17 primarily contributes to immunopathology, increased production of IL-13 and increased mucus production in the lungs [21, 23, 27, 28, 41]; although these effects vary depending upon the virus strain used. Importantly, conclusions drawn from rodent models of RSV must be interpreted with caution, as it is clear that mice are only semi-permissive to virus infection and do not reflect many aspects of disease pathogenesis and immunity observed in RSV-infected children [3, 5, 6]. As a fully-permissive host for BRSV, the calf model may be ideal for more clearly determining the role of IL-17 in immunity to RSV infection, as well as elucidating the circumstances that contribute to a protective or pathogenic role for IL-17.

Immunity in the lungs relies on a delicate balance between protective and pathogenic inflammation. Thus, it is likely that some production of IL-17 may contribute to host protection, but an imbalance in this response can be damaging. The development of BRDC is not clearly understood, but usually involves a primary viral infection leading to secondary bacterial pneumonia [7, 9, 12, 62]. In a recent report, Li *et al*. used a mouse model to demonstrate that primary influenza virus infection suppressed the production of IL-17 by murine γδ T cells, leading to increased susceptibility to secondary bacterial infection with *Streptococcus pneumonia* [63]. As a similar situation may be occurring in the calf with viral/bacterial co-infections, we hypothesized that primary BRSV infection would impair *M*. *haemolytica*-induced IL-17 production. In contrast, however, we observed significantly increased production of IL-17 in cell cultures exposed to both BRSV and *M*. *haemolytica* together, compared to either pathogen singly. It is important to note that our *in vitro* BRDC experiments were all carried out using lymphocytes isolated from peripheral blood. It is currently unknown if similarly exacerbated IL-17 production would be observed using cells isolated from the lungs, or during *in vivo* BRSV/*M*. *haemolytica* co-infection. It is possible that lung-resident subsets of CD4 and γδ T cells may respond differently to co-infection than cells from the blood, or that effects of the *in vivo* lung microenvironment may alter IL-17 production and the inflammatory response. However, given the significant neutrophilia that is observed in calves with BRDC, our *in vitro* experiments support the possibility that exacerbated IL-17 production, and hence exacerbated neutrophil recruitment, may be occurring during *in vivo* infection. Thus, based upon results from our *in vitro* coinfection model, we hypothesize that increased and exacerbated production of IL-17 in the lungs may contribute to the unbalanced immune response and disease observed in calves with BRDC, and suggest that γδ T cells may be critical to this outcome. In future experiments, we plan to test this hypothesis and determine the contribution of IL-17 production on disease pathogenesis *in vivo* in the BRDC affected calf.

*M*. *haemolytica* and *P*. *multocida* are closely related members of the family Pasteurellaceae and common causes of the secondary bacterial pneumonia observed in calves with BRDC [9,62]. Using our *in vitro* co-infection model of BRDC, we observed that *M*. *haemolytica* infection resulted in exacerbated IL-17 production, while secondary infection with *P*. *multocida* did not. Importantly, although *M*. *haemolytica* and *P*. *multocida* are in the same family, they are genetically quite different and induce a different pathologic outcome in the infected host [8, 9]. In particular, *M*. *haemolytica* induces a more acute infection, while pneumonia caused by *P*. *multocida* progresses more slowly. At this time we do not know the mechanism contributing to exacerbated IL-17 during BRSV/*M*. *haemolytica* coinfection, nor is it clear why *P*. *multocida* does not induce a similar response. One possible mechanism may be the action of *M*. *haemolytica’s* leukotoxin. Leukotoxin binds CD18 on ruminant leukocytes causing rapid, necrotic cell death, and is hypothesized to play a role in the inflammation and neutrophilia observed in cattle with *M*. *haemolytica* pneumonia [34]. *P*. *multocida* does not express leukotoxin [8, 9]. Further analysis of differential immune responses induced by these two pathogens *in vitro* and *in vivo* may contribute to our knowledge of host immunity and disease pathogenesis during BRDC in the calf.

In conclusion, with this report, we have demonstrated for the first time the production of IL-17, IL-21 and IL-22 in the calf with BRSV infection. We have also described a possible mechanism for IL-17 leading to exacerbated inflammation that is commonly observed during BRDC in the calf. Our results have implications for both children infected with RSV, and neonatal calves affected by respiratory disease. A more detailed analysis of the *in vivo* role of IL-17 in both contexts is critical to increase our understanding of disease pathogenesis for both RSV and BRDC. Further studies may also implicate IL-17 and Th17 responses as a critical target for therapeutic intervention in both children and cattle with RSV.
